# Real‐World Utilization Patterns of Toxoplasmosis Diagnostic Testing in Korea: A Large‐Scale Laboratory‐Based Study

**DOI:** 10.1002/jcla.70231

**Published:** 2026-04-21

**Authors:** Rihwa Choi, Sang Gon Lee

**Affiliations:** ^1^ Laboratory Medicine Center, GC Labs Yongin Gyeonggi Republic of Korea; ^2^ Department of Laboratory Medicine and Genetics Sungkyunkwan University School of Medicine Seoul Republic of Korea

**Keywords:** immunoglobulin G, immunoglobulin M, polymerase chain reaction, test utilization, toxoplasma

## Abstract

**Background:**

Toxoplasmosis is primarily diagnosed using serologic assays, while PCR is applied selectively in specific clinical contexts. However, data on real‐world utilization patterns of toxoplasmosis diagnostic tests remain limited, particularly in settings where the disease is relatively uncommon.

**Methods:**

We retrospectively analyzed laboratory data for *Toxoplasma gondii* PCR, IgG, and IgM assays performed for clinical purposes between January 2020 and December 2024 at a large referral laboratory in Korea. Test utilization patterns, test combinations, test results measured after the initial test, and factors associated with test positivity were evaluated using descriptive statistics and multivariable logistic regression analyses.

**Results:**

A total of 28,668 subjects underwent at least one toxoplasmosis‐related test. Serologic testing accounted for the majority of initial evaluations, whereas PCR was used infrequently and primarily as a context‐dependent adjunct. IgM positivity tended to be associated with subsequent testing after the initial test, while IgG positivity was more commonly observed with increasing age. Tests performed after the initial test were variably performed. PCR positivity suggestive of acute infection was rare and was observed only in cerebrospinal fluid and vitreous fluid specimens. Overall, testing patterns varied across institutions.

**Conclusions:**

This large‐scale study provides an overview of real‐world toxoplasmosis diagnostic test utilization in Korea. Considerable variation was observed in test utilization patterns, including IgM use and testing performed after the initial test. Further studies incorporating clinical information are needed to clarify the clinical context and implications of these patterns.

## Graphical abstract

We conducted a large retrospective analysis of real‐world Toxoplasma gondii IgG, IgM, and PCR testing performed at a nationwide referral laboratory in Korea to characterize diagnostic utilization patterns and test performance in routine clinical practice. Toxoplasmosis diagnostic practices in Korea show substantial variability, with serology predominating and PCR used selectively in specific clinical settings.

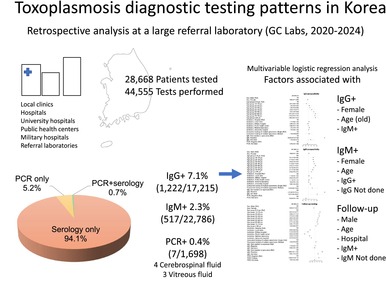

## 1 Introduction

Toxoplasmosis is a zoonotic parasitic infection caused by *Toxoplasma gondii* (*T. gondii*), an obligate intracellular protozoan with worldwide distribution [1, 2, 3]. Humans primarily acquire infection through ingestion of tissue cysts in undercooked meat or oocysts shed by felines, the definitive host of the parasite [1, 3]. Although toxoplasmosis is usually asymptomatic or causes only mild, self‐limited illness in immunocompetent individuals, infection during pregnancy or in immunocompromised hosts can result in severe and potentially life‐threatening disease [1, 2]. Primary infection during pregnancy may lead to congenital toxoplasmosis, which is associated with miscarriage, stillbirth, or long‐term sequelae such as chorioretinitis, hydrocephalus, and intracranial calcifications [1, 2, 4]. In individuals with impaired cellular immunity, particularly those with advanced human immunodeficiency virus (HIV) infection or other causes of immunosuppression, reactivation of latent infection may cause toxoplasmic encephalitis, a major cause of central nervous system morbidity [2, 5, 6]. Recently, the importance of diagnosing *T. gondii* infection has been increasingly emphasized in association with a wide range of neuropsychiatric disorders [2, 5, 6]. Because clinical manifestations are often nonspecific and a substantial proportion of infections remain subclinical, laboratory testing plays a central role in the diagnosis of toxoplasmosis [7, 8, 9, 10].

In Korea, toxoplasmosis is a Group 4 nationally notifiable infectious disease monitored through a sentinel surveillance system operated by the Korea Disease Control and Prevention Agency (KDCA), which primarily tracks temporal trends rather than true population incidence [9, 10, 11, 12]. Although national health insurance claims data using the KCD code B58 (toxoplasmosis) provide information on healthcare utilization, these data are limited by administrative coding and may include suspected or non–laboratory‐confirmed cases, and do not capture patterns of diagnostic test utilization [7, 13]. Moreover, in the Healthcare Big Data Open System of the Health Insurance Review and Assessment Service (HIRA), laboratory test utilization can be identified only by reimbursement codes, many of which are shared across multiple parasitic infections (e.g., Entamoeba, *Giardia lamblia* , Plasmodium species, and *Trichomonas vaginalis*) [13]. Consequently, toxoplasmosis‐specific serologic and nucleic acid amplification (PCR) tests utilization cannot be reliably distinguished using publicly available data, limiting insight into real‐world diagnostic practices in Korea [7, 13, 14, 15, 16, 17].

Current laboratory diagnosis of toxoplasmosis relies primarily on serologic assays detecting *Toxoplasma gondii*–specific IgG and IgM antibodies, with additional tests such as IgG avidity assays and nucleic acid amplification tests (PCR) used in selected clinical contexts, including pregnancy, congenital infection, and immunocompromised hosts [1, 8, 18, 19]. However, interpretation of serologic results—particularly IgM positivity—is complicated by prolonged antibody persistence, false‐positive reactions, and substantial inter‐assay variability, with a reported geometric coefficient of variation of up to 41.0% [18, 19, 20, 21, 22]. Accordingly, international guidelines emphasize the limitations of isolated IgM testing and recommend context‐dependent combinations of serologic assays or confirmatory testing, including PCR in appropriate specimens [1, 4, 6, 7, 8, 9, 10]. Acute toxoplasmosis is relatively uncommon and often asymptomatic, and diagnosis in routine clinical practice therefore relies mainly on serologic markers such as IgG, IgM, and IgG avidity [1, 2]. Consequently, PCR is used primarily as a complementary test in selected clinical settings, including suspected congenital infection and immunocompromised patients [6, 7, 8, 9, 10].

Despite these well‐recognized diagnostic challenges, data on real‐world utilization patterns of toxoplasmosis diagnostic tests in Korea remain limited. In particular, it is unclear how frequently PCR, IgG, and IgM assays are used in routine clinical practice. Accordingly, this study aimed to characterize utilization patterns of toxoplasmosis diagnostic testing in Korea by retrospectively analyzing clinically performed *Toxoplasma gondii*–related tests, including PCR, IgG, and IgM, at a large referral laboratory.

## 2 Methods

### 2.1 Participants

This retrospective study analyzed laboratory testing data obtained from the laboratory information system (LIS) of GC Labs, Korea. GC Labs is a large referral clinical laboratory providing *Toxoplasma gondii* IgG, IgM, and PCR testing services to local clinics, general hospitals, tertiary and university hospitals, military hospitals, and public health centers nationwide. All *T. gondii*–related PCR, IgG, and IgM test results requested for diagnostic purposes between January 1, 2020, and December 31, 2024, were included. Test records lacking information on age or sex and specimens with insufficient volume that could potentially compromise result reliability were excluded. The study was conducted in accordance with the Declaration of Helsinki. All data were fully anonymized prior to analysis, and individual subjects were not identifiable. The study protocol was reviewed and approved by the Institutional Review Board of GC Labs (GCL‐2025‐1054‐01), with a waiver of informed consent granted due to the retrospective design and the use of de‐identified data.

### 2.2 Analytical Procedures

Toxoplasma IgG and IgM were measured using automated immunoassays (Access Toxo IgG and IgM II) on the UniCel DxI 800 analyzer (Beckman Coulter, Brea, CA, USA) according to the manufacturer's instructions, and results were reported qualitatively. IgG results were interpreted as non‐reactive (< 7.5 IU/mL), equivocal (7.5–10.4 IU/mL), or reactive (≥ 10.5 IU/mL). IgM results were expressed as signal‐to‐cutoff ratios (S/CO) and interpreted as non‐reactive (S/CO < 0.8), grey‐zone (0.8–0.9), or reactive (≥ 1.0). *Toxoplasma gondii* PCR testing was performed on clinically indicated specimens using established methods [23, 24, 25], with results reported qualitatively as positive or negative. Nucleic acids were extracted using the MagNA Pure 96 system (Roche, Mannheim, Germany), followed by amplification on the Applied Biosystems 2720 Thermal Cycler (Thermo Fisher Scientific, Foster City, CA, USA) and analysis using the QIAxcel system (QIAGEN, Hilden, Germany). HotStarTaq Plus Master Mix or AllTag Master Mix Kit (QIAGEN) was used according to the study period, and internal quality control was performed using Toxoplasma gondii DNA Control (VIRCELL, Granada, Spain). PCR amplification of the surface antigen 1 (SAG1) gene, encoding the major surface antigen p30, was performed using a method reported to detect as few as 1–10 parasites per sample [26]. In addition, this assay demonstrated 100% detection at 100–200 copies/μL using low‐positive control material during the study period.

### 2.3 Statistical Methods

Descriptive statistics were used to summarize baseline characteristics, testing strategies, and laboratory results. Categorical variables are presented as counts and percentages. Continuous variables are summarized as median with interquartile range (IQR) for non‐normally distributed data, as appropriate. Characteristics were compared across test utilization patterns at the initial measurement (serology only, PCR only, and PCR plus serology) using the chi‐square test for categorical variables and analysis of variance (ANOVA) for continuous variables, according to data distribution. Multivariable logistic regression analyses were performed to evaluate factors associated with IgM positivity, IgG positivity, and subsequent testing. IgG, IgM, and DNA test results were modeled as three‐level categorical variables, with “not done” treated as a separate category to reflect real‐world test‐ordering behavior. This approach allowed inclusion of all subjects without introducing selection bias from complete‐case analysis. Equivocal or grey‐zone serologic results were classified as non‐reactive for multivariable logistic regression analyses, consistent with the approach used in previous toxoplasma serology studies [27]. For cases in which PCR and serologic tests were ordered concurrently, agreement analysis was conducted using PCR results as the comparator [28]. This comparison was conducted to describe test utilization and result patterns, rather than to evaluate clinical performance using clinical information. Because clinical information was limited, it was difficult to determine whether subsequent tests represented clinical follow‐up prompted by suspected acute infection or testing performed for unrelated reasons. Therefore, all tests performed after the initial test were described as subsequent tests. For longitudinal analyses, serologic conversion was defined as a change from IgM non‐reactive or grey‐zone at the initial test to IgM reactive at any subsequent test result after the initial test. All statistical analyses were performed MedCalc Statistical Software version 23.3.7 (MedCalc Software Ltd., Ostend, Belgium; https://www.medcalc.org; 2025) and R software (version 4.5.2; R Foundation for Statistical Computing, Vienna, Austria). A *p*‐value < 0.05 was considered statistically significant.

## 3 Results

### 3.1 Study Population and Characteristics at the Initial Measurement

During the 5‐year study period, a total of 28,668 subjects underwent at least one toxoplasmosis‐related test (PCR, IgG, or IgM), yielding 44,555 tests in total. The study population was predominantly female (66.0%, 18,916/28,668), with a median age of 32.3 years (IQR, 23.0–41.7). Adults aged 20–39 years accounted for more than half of the study population (50.7%, 14,553/28,668), whereas children aged 0–9 years comprised 19.2% (5500/28,668) of subjects (Table 1). Initial testing was most frequently requested by university hospitals (39.8%, 11,421/28,668) and general hospitals (30.1%, 8625/28,668). Most subjects (95%, 27,243/28,668) underwent a single toxoplasmosis‐related test, while the remaining 5.0% (1425/28,668) underwent subsequent testing after the initial test.

**TABLE 1:** Baseline characteristics of subjects undergoing toxoplasmosis testing.

Characteristic	Overall (*N* = 28,668)
*Sex*	
Male	9752 (34.0)
Female	18,916 (66.0)
*Age, years*	
Median (interquartile range)	32.3 (23.0–41.7)
*Age group, years*	
0–9	5500 (19.2)
10–19	867 (3.0)
20–29	5146 (18.0)
30–39	9387 (32.7)
40–49	2301 (8.0)
50–59	2066 (7.2)
60–69	1852 (6.5)
70–79	1031 (3.6)
≥ 80	518 (1.8)
*Institution type*	
General hospital	8625 (30.1)
University hospital	11,421 (39.8)
Local clinic	4704 (16.4)
Public health center	1711 (6.0)
Referral laboratory	1866 (6.5)
Military hospital	341 (1.2)
*Number of subsequent tests per subject*	
0 (no subsequent tests)	27,243 (95.0)
1	1111 (3.9)
2	222 (0.8)
3	63 (0.2)
4	23 (0.1)
≥ 5	6 (< 0.1)

*Note:* Values are presented as *n* (%) unless otherwise indicated. Subjects were included if at least one toxoplasmosis‐related test (PCR, IgG, or IgM) was performed at the initial measurement.

### 3.2 Test Utilization Patterns at the Initial Measurement

Of the 28,668 subjects, 27,243 had no subsequent tests after the initial assessment, whereas 1425 underwent subsequent testing. All possible combinations of the initial test orders for IgG, IgM, and PCR were first identified, and subjects were subsequently categorized into broader testing pattern groups. Test combinations at the initial measurement included IgG only, IgM only, IgG plus IgM, PCR only, PCR plus IgG, PCR plus IgM, and PCR plus IgG and IgM. The majority of subjects underwent serology‐only testing (94.1%, 25,645/27,243), while 5.2% (1406/27,243) received PCR‐only testing and 0.7% (192/27,243) underwent combined PCR and serologic testing.

Table 2 summarizes test utilization patterns and positivity rates based on 39,666 specimens obtained from 27,243 subjects without subsequent tests after the initial measurement during the 5‐year study period. Among these subjects, 14,939 underwent a single test (PCR, IgG, or IgM), and 50.4% (7536/14,939) of these requests originated from university hospitals. A total of 12,192 subjects underwent two tests at the initial measurement without subsequent tests. Among them, 24 subjects had two specimens submitted for the same PCR test; except for one case referred from a general hospital, all remaining cases were referred from university hospitals. The remaining 12,163 subjects underwent two different tests, of whom 12,072 (99.0%) were tested exclusively with a combination of serologic assays (IgG and IgM). Overall, test positivity was most frequently observed for IgG among test combinations including IgG, followed by IgM and PCR. When multiple tests were ordered concurrently, the highest positivity rate was observed in cases in which PCR, IgG, and IgM were all requested; however, all PCR results in this group were negative. Among subjects without subsequent testing after the initial measurement, PCR positivity was identified only in six cases in which PCR was ordered without accompanying serologic tests; three were referred from university hospitals and three from other referral laboratories.

**TABLE 2:** Test utilization pattern for 27,243 subjects without subsequent testing (test only once).

Test combination at the initial test (no subsequent tests)	Number of subjects	Results with positive/reactive (+)	Results with equivocal/grey‐zone (±)
Test combination (one test per subject)	14,939 (54.8)	493 (3.3)	63 (0.5)
PCR only	1379 (9.2)	5 (0.4)	N/A
IgG only	3922 (26.3)	362 (9.2)	34 (0.9)
IgM	9638 (64.5)	126 (1.3)	29 (0.3)
Test combination (2 tests per subject)	12,192 (44.8)	771 (6.3)	85 (0.7)
PCR and PCR	24 (0.2)	1 (4.2)	N/A
IgG and IgG	2 (< 0.1)	0 (0.0)	0 (0.0)
IgM and IgM	3 (< 0.1)	0 (0.0)	0 (0.0)
IgG and IgM	12,072 (99.0)	759 (6.3) 29 IgG+/IgM± 477 IgG+/IgM– 59 IgG–/IgM+ 194 IgG+/IgM+	85 (0.7)30 IgG–/IgM±55 IgG±/IgM—
PCR and IgG	71 (0.6)	10 (14.1) 10 PCR–/IgG+	0 (0.0)
PCR and IgM	20 (0.2)	1 (4.2) 1 PCR–/IgM+	0 (0.0)
Test combination (3 tests per subject)	105 (0.4)	12 (11.4)	1 (1.0)
PCR, IgG, and IgM	97 (92.4)	12 (32.4) 1 PCR–/IgG±/IgM+ 5 PCR–/IgG+/IgM– 6 PCR–/IgG+/IgM+	1 (1.0)1 PCR–/IgG±/IgM—
PCR, PCR, and IgG	1 (1.0)	0 (0.0)	0 (0.0)
IgG, IgM, and IgM	3 (2.9)	0 (0.0)	0 (0.0)
PCR, PCR, and IgM	1 (1.0)	0 (0.0)	0 (0.0)
PCR, PCR, and PCR	3 (2.9)	0 (0.0)	N/A
Test combination (4 tests per subject)	7 (< 0.1)	0 (0.0)	0 (0.0)
IgG, IgG, IgM, and IgM	5 (71.4)	0 (0.0)	0 (0.0)
PCR, PCR, IgG, and IgM	1 (14.3)	0 (0.0)	0 (0.0)
PCR, PCR, PCR, and IgM	1 (14.3)	0 (0.0)	0 (0.0)
Test combination (5 tests per subject)	0 (0.0)	N/A	N/A
PCR, IgG, IgG, IgM, and IgM	0 (0.0)	N/A	N/A

*Note:* Values are presented as *n* (%) unless otherwise indicated. –, indicates a negative result for the DNA PCR test or a non‐reactive result for IgG or IgM tests; ±, indicates an equivocal or grey‐zone result for IgG or IgM tests; and +, indicates a positive result for the DNA PCR test or a reactive result for IgG or IgM tests. Because PCR test results are reported only as positive or negative, equivocal or grey‐zone results are not applicable to PCR tests.

Abbreviation: N/A, not applicable.

Test results at the initial measurement for the 1425 subjects with subsequent testing are presented in Table 3. The median interval from the initial tests to subsequent testing was 195 days (IQR, 49–705 days). Most subjects (99.2%, 1414/1425) underwent two or fewer tests at the initial measurement, and their positivity rates were higher than those of subjects without subsequent testing. Table 4 summarizes characteristics at the initial measurement stratified by subsequent testing status and test positivity for each assay. Age group and institution type differed significantly according to both subsequent testing status and test positivity (all *p* < 0.05). In contrast, sex was significantly associated with subsequent testing status and IgG positivity (both *p* < 0.01) but not with IgM or PCR positivity.

**TABLE 3:** Test utilization pattern for 1425 subjects with subsequent testing.

Test combination at the initial test (no subsequent test)	Number of subjects	Results with positive/reactive (+)	Results with equivocal/grey‐zone (±)
Test combination (one test per subject)	767 (53.7)	78 (9.8)	16 (2.1)
PCR only	76 (9.5)	1 (1.3)	N/A
IgG only	392 (49.2)	37 (9.4)	3 (0.8)
IgM	299 (37.5)	40 (13.4)	13 (4.3)
Test combination (2 tests per subject)	647 (45.3)	134 (20.7)	18 (2.8)
PCR and PCR	5 (0.8)	0 (0.0)	N/A
IgG and IgG	1 (0.2)	0 (0.0)	0 (0.0)
IgM and IgM	0 (0.0)	N/A	N/A
IgG and IgM	633 (97.8)	131 (20.7) 8 IgG+/IgM± 37 IgG+/IgM– 33 IgG–/IgM+ 51 IgG+/IgM+ 2 IgG±/IgM+	18 (2.8)15 IgG–/IgM±3 IgG±/IgM—
PCR and IgG	5 (0.8)	1 (20.0)	0 (0.0)
PCR and IgM	3 (0.5)	2 (66.7)	0 (0.0)
Test combination (3 tests per subject)	10 (0.7)	2 (20.0)	1 (10.0)
PCR, IgG, and IgM	9 (90.0)	2 (22.2) 1 PCR–/IgG–/IgM+ 1 PCR–/IgG+/IgM+	1 (11.1) 1 PCR–/IgG–/IgM±
PCR, PCR, and IgG	1 (10.0)	1 (100.0)	0 (0.0)
IgG, IgM, and IgM	0 (0.0)	N/A	N/A
PCR, PCR, and IgM	0 (0.0)	N/A	N/A
PCR, PCR, and PCR	0 (0.0)	N/A	N/A
Test combination (4 tests per subject)	0 (0.0)	0 (0.0)	0 (0.0)
IgG, IgG, IgM, and IgM	0 (0.0)	0 (0.0)	0 (0.0)
PCR, PCR, IgG, and IgM	0 (0.0)	N/A	N/A
PCR, PCR, PCR, and IgM	0 (0.0)	N/A	N/A
Test combination (5 tests per subject)	1 (0.1)	1 (100.0)	0 (0.0)
PCR, IgG, IgG, IgM, and IgM	1 (100.0)	1 (100.0)	0 (0.0)

*Note:* Values are presented as *n* (%) unless otherwise indicated. –, indicates a negative result for the DNA PCR test or a non‐reactive result for IgG or IgM tests; ±, indicates an equivocal or grey‐zone result for IgG or IgM tests; and +, indicates a positive result for the DNA PCR test or a reactive result for IgG or IgM tests. Because PCR test results are reported only as positive or negative, equivocal or grey‐zone results are not applicable to PCR tests.

Abbreviation: N/A, not applicable.

**TABLE 4:** Differences in characteristics according to subsequent testing status and test positivity.

Characteristics	Total subjects (*n* = 28,668)	Subsequent tests			IgG reactivity			IgM reactivity			DNA PCR positivity		
		No subsequent tests (*n* = 27,243)	Subsequent tests (*n* = 1425)	*p*	IgG– (*n* = 15,993)	IgG+(*n* = 1222)	*p*	IgM–(*n* = 22,269)	IgM+(*n* = 517)	*p*	PCR–(*n* = 1691)	PCR+(*n* = 7)	*p*
Sex				0.0013			< 0.0001			0.3636			0.9692
Male	9752 (34.0)	9211 (33.8)	541 (38.0)		4547 (28.4)	491 (40.2)		6525 (29.3)	161 (31.1)		737 (43.6)	3 (42.9)	
Female	18,916 (66.0)	18,032 (66.2)	884 (62.0)		11,446 (71.6)	731 (59.8)		15,744 (70.7)	356 (68.9)		954 (56.4)	4 (57.1)	
Age group				< 0.0001			< 0.0001						0.0661
0–9 years	5500 (19.2)	5415 (19.9)	85 (6.0)		273 (1.7)	8 (0.7)		5268 (23.7)	43 (8.3)		162 (9.6)	0 (0.0)	
10–19 years	867 (3.0)	797 (2.9)	70 (4.9)		456 (2.9)	12 (1.0)		625 (2.8)	14 (2.7)		65 (3.8)	1 (14.3)	
20–20 years	5146 (18.0)	4852 (17.8)	294 (20.6)		3458 (21.6)	148 (12.1)		4077 (18.3)	81 (15.7)		212 (12.5)	0 (0.0)	
30–39 years	9387 (32.7)	8846 (32.5)	541 (38.0)		7100 (44.4)	347 (28.4)		7965 (35.8)	170 (32.9)		353 (20.9)	0 (0.0)	
40–49 years	2301 (8)	2165 (7.9)	136 (9.5)		1541 (9.6)	133 (10.9)		1426 (6.4)	52 (10.1)		189 (11.2)	0 (0.0)	
50–59 years	2066 (7.2)	1941 (7.1)	125 (8.8)		1285 (8.0)	195 (16.0)		1070 (4.8)	75 (14.5)		226 (13.4)	0 (0.0)	
60–69 years	1852 (6.5)	1757 (6.5)	95 (6.7)		1024 (6.4)	219 (17.9)		960 (4.3)	53 (10.3)		257 (15.2)	3 (42.9)	
70–79 years	1031 (3.6)	971 (3.6)	60 (4.2)		560 (3.5)	103 (8.4)		569 (2.6)	20 (3.9)		158 (9.3)	2 (28.6)	
≥ 80 years	518 (1.8)	499 (1.8)	19 (1.3)		296 (1.9)	57 (4.7)		309 (1.4)	9 (1.7)		69 (4.1)	1 (14.3)	
Institution type				< 0.0001			< 0.0001			0.0003			0.3236
General hospital	8625 (30.1)	7877 (28.9)	748 (52.5)		4272 (26.7)	318 (26.2)		7163 (32.2)	127 (24.6)		73 (4.3)	0 (0.0)	
Local clinic	4704 (16.4)	4560 (16.7)	144 (10.1)		3521 (22.0)	191 (15.6)		4352 (19.5)	97 (18.8)		40 (2.4)	0 (0.0)	
Military hospital	341 (1.2)	333 (1.2)	8 (0.6)		308 (1.9)	16 (1.3)		310 (1.4)	10 (1.9)		0 (0.0)	0 (0.0)	
Public health center	1711 (6.0)	1699 (6.2)	12 (0.8)		1526 (9.5)	68 (5.6)		1558 (7.0)	30 (5.8)		2 (0.1)	0 (0.0)	
Referral laboratory	1866 (6.5)	1812 (6.7)	54 (3.8)		1035 (6.5)	83 (6.8)		1282 (5.8)	30 (5.8)		247 (14.6)	3 (42.9)	
University hospital	11,421 (39.8)	10,962 (40.2)	459 (32.3)		5331 (33.3)	546 (44.7)		7604 (34.2)	223 (43.1)		1329 (78.6)	4 (57.1)	

*Note:* Values are presented as *n* (%) unless otherwise indicated. –, indicates a negative result for the DNA PCR test or a non‐reactive result for IgG or IgM tests and +, indicates a positive result for the DNA PCR test or a reactive result for IgG or IgM tests. Equivocal or grey‐zone results were classified as non‐reactive in the analysis.

**FIGURE 1:**
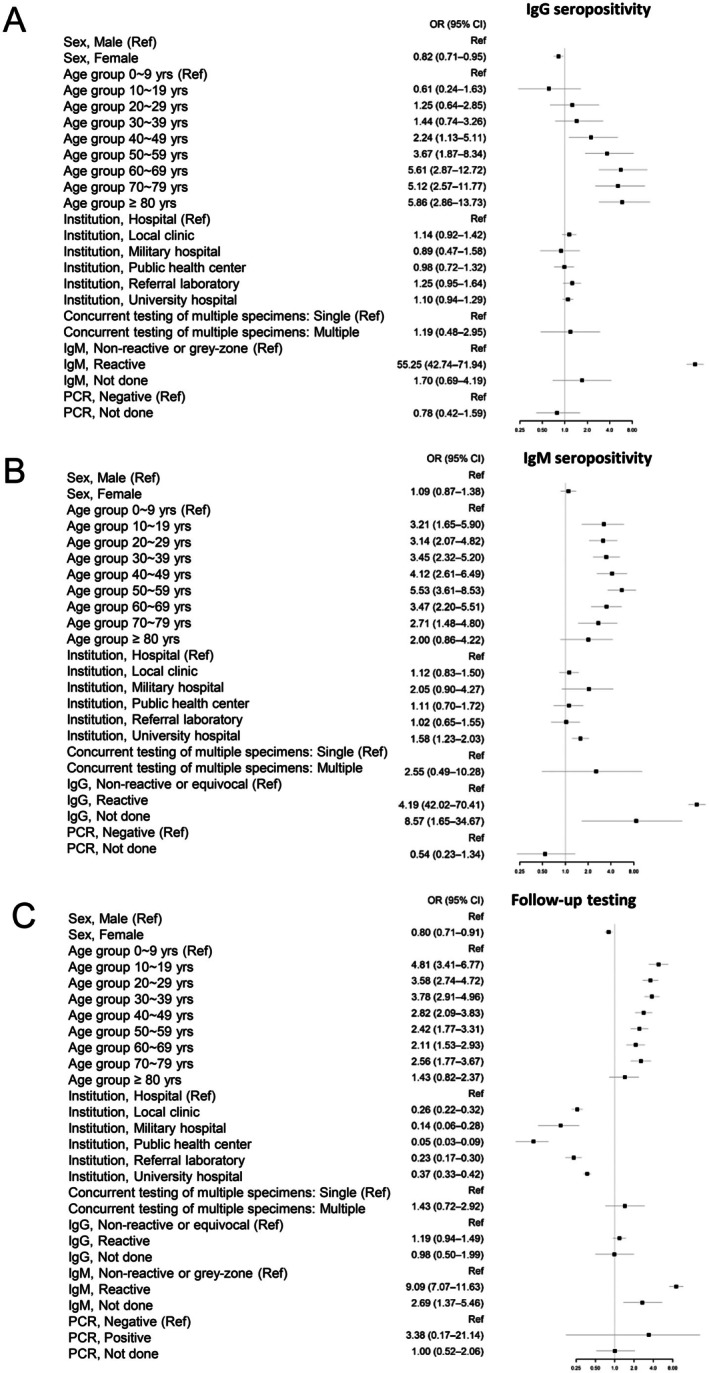
Forest plots of multivariable logistic regression analyses for IgG seropositivity (A), IgM seropositivity (B), and subsequent testing (C). Odds ratios (ORs) with 95% confidence intervals (CIs) are presented for each variable. Reference categories are indicated in each panel and shown as OR = 1.00. Equivocal or grey‐zone results were classified as non‐reactive in the analysis.

In multivariable logistic regression analyses, IgM and IgG positivity showed a strong association (Figure 1). IgM positivity was most strongly associated with subsequent testing, indicating that IgM‐reactive results frequently prompted subsequent testing. IgG positivity was primarily associated with increasing age and IgM positivity, consistent with cumulative exposure patterns. Subsequent testing was independently associated with IgM positivity, IgG positivity, age group, sex, and institution type, highlighting the combined influence of serologic results and clinical testing practices. Compared with hospitals, subsequent testing was significantly less frequent in other healthcare settings.

At the initial request, 1739 PCR results from 1698 subjects who underwent DNA PCR testing were identified, with most subjects (97.8%, 1661/1698) submitting a single specimen. PCR positivity rates differed significantly by specimen type (chi‐square test, *p* = 0.0001). Whole blood was the most frequently requested specimen (57.2%, 994/1739), followed by cerebrospinal fluid (24.5%, 426/1739), amniotic fluid (7.5%, 131/1739), and vitreous fluid (5.5%, 96/1739). PCR‐positive results were observed only in cerebrospinal fluid (*n* = 3) and vitreous fluid (*n* = 4) from seven subjects, all of which were requested by university hospitals (*n* = 4) or other referral laboratories (*n* = 3).

### 3.3 Subsequent Testing After the Initial Measurement

When grey‐zone (equivocal) results were classified as negative, 1208 of the 1425 subjects who underwent subsequent testing had no positive results for PCR, IgG, or IgM at the initial assessment. Among these subjects, 18 subsequently showed positive results in tests performed after the initial test. Of these 18 subjects with newly positive findings, 11 demonstrated seroconversion from negative to positive. Among the remaining seven subjects, six had IgM results in the grey zone at the initial test that later became positive in tests performed after the initial test; additional PCR testing was performed in these cases and yielded negative results. One subject showed a change from equivocal IgG to positive IgG in tests performed after the initial test. The interval from the initial tests to subsequent testing among subjects with an initial IgM grey‐zone result was significantly shorter, with a median of 14.5 days (IQR, 13–20 days), compared with a median interval of 558.5 days (IQR, 237.5–1490 days) among subjects without an initial IgM grey‐zone result (*p* = 0.0066). Among the 1425 subjects who underwent subsequent testing, 131 had IgM‐positive results at the initial assessment, and all remained IgM positive in tests performed after the initial test. The interval from the initial tests to subsequent testing for these subjects ranged from 2 to 1481 days (maximum, 4.1 years), with a median of 16 days (IQR, 7–58 days). Among these 131 subjects, 76 (58.0%) underwent DNA PCR testing either concurrently or subsequently, and PCR positivity was identified in one subject (1.3%, 1/76).

### 3.4 Agreement Between Baseline IgM Serology and Toxoplasma PCR Results

Among all subjects, 108 had results available for all three assays (PCR, IgG, and IgM) at the initial assessment. Using the initial test results from these subjects, a 2 × 2 contingency table was constructed with PCR results serving as the comparator to evaluate agreement between the IgM antibody assay and PCR. However, no PCR‐positive results were observed among these 108 test sets; therefore, positive percent agreement could not be calculated. The overall agreement was 91.7% (99/108, 95% confidence interval [CI], 84.9%–95.6%), which was identical to the negative percent agreement (Table 5). The results table included one subject with IgG equivocal/IgM negative/PCR negative results, one subject with IgG negative/IgM grey‐zone/PCR negative results, and one subject with IgG positive/IgM grey‐zone/PCR negative results. When these cases were excluded from the analysis, the overall agreement (negative percent agreement) between the PCR assay and the IgM assay was 91.4% (96/105, 95% CI, 84.5%–95.4%). When equivocal or grey‐zone results were classified as negative, the overall agreement was 91.7% (99/108, 95% CI, 84.9%–95.6%), whereas classification of equivocal or grey‐zone results as positive yielded an overall agreement of 89.8% (97/108, 95% CI, 82.7%–94.2%).

**TABLE 5:** Toxoplasma PCR and serologic results at the initial measurement: 2 × 2 contingency analysis (*n* = 108).

IgM	PCR		Total	Results pattern			
	Negative	Positive		PCR–/IgG–/IgM–	PCR–/IgG–/IgM+	PCR–/IgG+/IgM–	PCR–/IgG+/IgM+
Non‐reactive	99	0	99 (91.7%)	92 (85.2)	0 (0.0)	7 (6.5)	0 (0.0)
Reactive	9	0	9 (8.3%)	0 (0.0)	1 (0.9)	0 (0.0)	8 (7.4)
Total	108	0	108 (100.0)	92 (85.2)	1 (0.9)	7 (6.5)	8 (7.4)

*Note:* Equivocal test results were considered negative for the purpose of analysis. –, indicates a negative result for the DNA PCR test or a non‐reactive result for IgG or IgM tests; and +, indicates a positive result for the DNA PCR test or a reactive result for IgG or IgM tests. Equivocal or grey‐zone results were classified as non‐reactive in the analysis. This table shows the agreement of test result patterns at the initial measurement. The agreement analysis between IgM and PCR is of limited interpretability because no PCR‐positive cases were observed among the analyzed samples. Therefore, the reported agreement reflects only negative concordance. Because no clinical information was available, these findings do not represent the diagnostic performance of the assays and should be interpreted with caution.

## 4 Discussion

In this retrospective study, we analyzed real‐world utilization patterns of diagnostic tests for toxoplasmosis, including PCR, IgG, and IgM assays, using 5 years of laboratory data from a large clinical laboratory in Korea.

In this study, subsequent testing after the initial measurement encompassed all tests performed later and therefore showed a wide range of intervals. Because it was not possible to determine whether such subsequent testing was prompted by clinical suspicion, the findings should be interpreted with caution. International guidelines from the U.S. CDC, the American College of Obstetricians and Gynecologists, and other expert bodies consistently emphasize that isolated IgM positivity is insufficient to establish recent infection and should be interpreted in conjunction with IgG kinetics, IgG avidity testing, or confirmatory testing performed at reference laboratories [4, 8, 9, 10, 14, 15, 16, 17, 18].

Meanwhile, PCR testing was used far less frequently than serologic assays. This finding may reflect its role as a targeted, context‐dependent diagnostic tool rather than a screening test, consistent with recommendations for specific clinical scenarios such as suspected congenital infection, immunocompromised hosts, or central nervous system involvement [4, 8, 9, 10]. The limited and selective use of PCR may reflect both its intrinsic characteristics—high specificity with variable sensitivity depending on specimen type and disease stage—and practical constraints, including uncertainty regarding optimal timing and specimen selection, higher costs, and limited availability of molecular diagnostic infrastructure [4, 15, 16]. In this study, PCR positivity was observed only in cerebrospinal fluid and vitreous fluid specimens, whereas no positive results were detected in whole blood, the most commonly submitted specimen. This pattern is consistent with the context‐dependent role of Toxoplasma gondii PCR, which is generally considered most useful in selected clinical settings, including central nervous system and ocular disease [1, 7, 8]. By contrast, the diagnostic yield of blood PCR is variable and may be influenced by parasite burden, timing of sampling, immune status, and treatment [1, 5, 6, 7, 8, 9, 10]. However, because clinical information on the reason for ordering PCR was not available in this study, the actual reason could not be determined, and the findings should therefore be interpreted with caution.

In very early infection, as well as in immunocompromised individuals and in fetuses or neonates with immature immune responses, IgM may remain negative while PCR yields positive results [1, 6, 8]. Conversely, IgM positivity accompanied by high IgG avidity and negative PCR findings is more consistent with past infection than with active disease [1, 8, 21]. Therefore, the diagnostic indications for PCR and serologic assays differ substantially, and these methods should not be considered interchangeable [1, 6, 8]. Accordingly, agreement analyses between PCR and serology should be interpreted with caution.

Notably, some university hospitals referred all three assays—IgG, IgM, and PCR—to GC Labs, underscoring the important role of centralized laboratories in settings where toxoplasmosis testing is relatively infrequent [29]. GC Labs provides specialized diagnostic services for rare and infrequent infectious diseases, many of which are difficult to sustain even in tertiary hospitals because of low testing volumes and technical complexity [30, 31]. In this context, referral laboratories play a critical role in ensuring access to specialized diagnostics for uncommon and technically demanding infections, highlighting the need for sustained institutional and policy‐level support rather than reimbursement‐driven disincentives [29, 30, 31].

This study has several limitations. First, as a laboratory‐based analysis, we lacked detailed clinical information—such as pregnancy status, immune status, clinical manifestations, and treatment decisions—which limited our ability to fully assess the appropriateness of individual testing decisions. The absence of detailed clinical data limits patient stratification and precludes a more precise clinical interpretation of PCR and serologic test utilization patterns; therefore, the findings of the present study should be interpreted with caution. Second, test utilization patterns observed at a single referral laboratory may not be fully generalizable to all healthcare settings in Korea, particularly smaller institutions or primary care clinics. Third, clinical outcomes were not evaluated; therefore, we could not directly link testing patterns to diagnostic accuracy or patient prognosis. In addition, in cases with IgM positivity, IgG avidity testing is helpful for distinguishing recent from past infection [1, 4, 8, 9]. The absence of IgG avidity assessment in the present study is a limitation in the interpretation of IgM‐positive results, particularly in the diagnosis of acute infection and related clinical decision‐making. Unfortunately, *Toxoplasma gondii* IgG avidity testing was not available for analysis in this study. According to HIRA data, IgG avidity testing is performed in only a limited number of hospitals in Korea, with only about 300 individuals undergoing the test annually, despite its established role in differentiating acute from chronic toxoplasmosis [13, 32, 33]. An additional limitation of this study is that equivocal/grey‐zone IgG and IgM results were classified as negative in the regression analyses to enable binary classification of test positivity [27]. Although alternative classifications of these results could be considered, the number of equivocal/grey‐zone cases was very small in our dataset, limiting the interpretability and potential value of reclassification‐based sensitivity analyses. Therefore, the findings should be interpreted with caution. Despite these limitations, this study provides an overview of toxoplasmosis diagnostic test utilization in Korea based on large‐scale laboratory data. Future studies integrating laboratory findings with clinical and administrative datasets may further clarify how diagnostic testing for toxoplasmosis could be optimized to improve clinical outcomes.

In conclusions, this large‐scale laboratory‐based study describes real‐world patterns of toxoplasmosis diagnostic testing in Korea. Serologic assays, including IgG and IgM, appear to constitute the main diagnostic approach, whereas PCR is used more selectively as a context‐dependent adjunct. IgM positivity tended to be associated with subsequent testing, while IgG positivity was more commonly observed with increasing age. Considerable variation was observed in test utilization patterns. Further studies incorporating clinical information are needed to clarify the clinical context and implications of these patterns.

## Author Contributions

All authors contributed to manuscript preparation. Conceptualization, data curation, investigation, methodology, formal analysis, validation, visualization: Rihwa Choi. Supervision, funding acquisition: Sang Gon Lee. Writing – original draft: Rihwa Choi. Writing – review and editing: Rihwa Choi and Sang Gon Lee. All authors read and approved the final manuscript.

## Funding

The authors have nothing to report.

## Ethics Statement

This study was conducted in accordance with the Declaration of Helsinki. The study protocol was reviewed and approved by the Institutional Review Board of GC Labs (GCL‐2025‐1054‐01).

## Consent

Waiver of informed consent granted due to the retrospective design and the use of de‐identified data.

## Conflicts of Interest

The authors declare no conflicts of interest.

## Data Availability

The datasets generated and analyzed during the current study are available from the corresponding authors on reasonable request.
